# Abdominal Hollowing vs. Abdominal Bracing: A Scoping Review of Clinical Trials on Effectiveness for Trunk Stability and Rehabilitation

**DOI:** 10.3390/jfmk9040193

**Published:** 2024-10-10

**Authors:** Iva Golob, Manca Opara Zupančič, Žiga Kozinc

**Affiliations:** Faculty of Health Sciences, University of Primorska, Polje 42, SI-6310 Izola, Slovenia; 97230366@student.upr.si (I.G.); manca.opara@fvz.upr.si (M.O.Z.)

**Keywords:** core stability, muscle activation, pain management, functional outcomes, clinical trials

## Abstract

**Objectives:** This scoping review explores the effectiveness of abdominal hollowing (AH) and abdominal bracing (AB) techniques in enhancing trunk stability and facilitating rehabilitation, particularly for individuals with lower back pain (LBP). **Methods:** The review synthesizes findings from 22 randomized controlled trials (RCTs) that assessed these techniques’ impacts on muscle activation, pain reduction, and functional outcomes. **Results:** The results demonstrate that both techniques can significantly improve trunk stability, muscle thickness, balance, and gait. However, a notable gap exists in studies directly comparing AH and AB, raising questions about whether they are equally effective. While AH is often associated with selective activation of the transversus abdominis, AB promotes a broader co-contraction of trunk muscles, contributing to robust spinal stability. **Conclusions:** This review underscores the need for further research to directly compare these techniques and refine their application in clinical practice. The findings suggest that personalized rehabilitation programs incorporating both AH and AB, tailored to individual patient needs and rehabilitation goals, can be effective in managing and preventing LBP.

## 1. Introduction

In contemporary society, the prevalence of low back pain (LBP) is notably high, posing significant challenges to individuals’ daily functioning and overall well-being. Not only does LBP hinder routine activities but it also correlates with diminished quality of life and potential psychological ramifications such as anxiety and depression [1]. LBP manifests across a spectrum ranging from acute (lasting less than six weeks), subacute (lasting six to twelve weeks), to chronic (lasting more than twelve weeks) [2]. A global survey conducted in 2017 revealed a considerable LBP prevalence of 7.5%, with approximately 42.5 million individuals experiencing associated disability. Notably, disability duration peaked between the ages of 45 to 49 years before declining [3].

Poor lumbar stability can lead to an imbalance in the muscles that support the spine, causing the lower back to be more vulnerable to injury. This can result in LBP, as well as other issues such as sciatica, herniated discs, and spinal stenosis [4]. The musculature of the abdominal wall plays a pivotal role in spinal support and stabilization [5]. Comprising the rectus abdominis (RA) at the midline and the external oblique (EO), internal oblique (IO), and transversus abdominis (TrA) laterally, these muscles form overlapping layers around the spine [6]. Collaboratively with the diaphragm, pelvic floor muscles, and lumbar multifidus, these muscles operate in synchronization to distribute internal and external trunk loads while regulating abdominal pressure [5]. By synergizing the activation of the deep abdominal muscles and the extensor muscles of the trunk, trunk stabilization acts like a supportive trunk corset to alleviate existing LBP and prevent its recurrence [7]. Exercise based on this kind of muscle activation not only reduces the dysfunction resulting from lumbar instability but also strengthens the adjacent muscles of the lower back [8].

Lumbar stability is important because it enables the maintenance of spinal neutral zones during routine functional activity without causing a neurological deficit, incapacitating pain, or significant deformity [4]. Exercises for lumbar stabilization have grown in popularity as an LBP treatment [8]. Training techniques for improving lumbar stabilization vary from employing weight machines made to strengthen the spine’s primary movers to training the multifidus and transversus abdominis (TrA) with isometric contraction [9,10].

LBP stabilizer training has evolved significantly over the years. Initially, the focus was on the general strengthening of the back muscles, but later research emphasized the importance of specific muscles, such as the TrA and multifidus, in stabilizing the spine [11]. In the 1990s, exercises became more targeted at these deep stabilizers [12]. Modern approaches combine specific exercises to activate these muscles with mobility improvement and functional exercises. Key techniques that allow for better spinal stabilization during daily activities and have become an integral part of the management of LBP include the abdominal hollowing (AH) and abdominal bracing (AB) techniques [13].

The AH exercise selectively engages the TrA muscle while minimizing the activation of global muscles such as the RA [14,15]. This method is particularly effective for activating muscles that contribute to the stability of the spine, specifically the RA, EO, IO, and TrA [16]. When performing AH in hook-lying, standing, sitting, or 4-point kneeling position, the patient should breathe in and out and then gently and slowly draw in their lower abdomen below the umbilicus without moving their upper stomach, back, and pelvis [14]. Studies have demonstrated that in individuals with non-specific LBP, the AH technique effectively reduces pain and improves low back disability [15,16,17]. Conversely, the AB technique involves simultaneous contraction of the RA, EO, TrA, and IO muscles in the anterolateral abdomen [17]. AB is a valuable strategy in bolstering lumbar stability [18]. Through the activation of the deep abdominal muscles, notably the TrA and IO muscles, along with the elevation of intra-abdominal pressure, AB facilitates an enhancement in spinal stabilization [1]. When performing AB, the patient should breathe in and out, and then gently and slowly push out their waist without drawing their abdomen inward or moving their back or pelvis [14]. The AB technique can be performed in a variety of positions such as supine, sitting, and standing and while performing functional movements such as pull-ups or squats. The progression of this technique includes a basic level by lying on the back with activation of the TrA muscles, an intermediate level by adding leg or arm movements while maintaining muscle activation, and an advanced level by performing complex movements such as planking or stabilisation exercises on unstable surfaces [19].

The optimal alignment of the lumbar spine, characterized by a neutral position, can be effectively attained through the integration of the AH and AB techniques, complemented by lumbar stabilization exercises. This approach is vital as the activation of muscles such as the IO, EO, and TrA collectively plays a significant role in enhancing spinal stability [20]. In summation, understanding the complexities of LBP and its impact on individuals’ lives underscores the necessity for effective therapeutic interventions. Given the integral role of abdominal musculature in spinal support and stabilization, techniques such as AH and AB have garnered attention for their potential to alleviate LBP and enhance spinal stability.

This literature review aims to explore the effectiveness of the AH and AB techniques. By synthesizing existing research, this review aims to provide insights into the practical application of these techniques and their implications for clinical practice and rehabilitation. The need for direct comparisons between AH and AB is particularly critical due to the biomechanical differences between the techniques. AH primarily targets deep muscles, such as the transversus abdominis, for selective activation, while AB promotes co-contraction of both deep and superficial muscles, including the obliques and rectus abdominis. These differing muscle activation patterns suggest that AH and AB may offer distinct benefits depending on the patient’s condition and rehabilitation goals. Thus, this review aims to address this gap in the literature by summarizing current evidence and advocating for further research to directly compare these techniques in clinical practice.

## 2. Materials and Methods

This scoping review examines the effectiveness of abdominal hollowing AH and AB techniques for trunk stability and rehabilitation. The methodology followed the PRISMA extension for Scoping Reviews (PRISMA-ScR) guidelines [21]. This scoping review was not registered with PROSPERO, as scoping reviews are currently not eligible for registration in PROSPERO.

### 2.1. Study Eligibility Criteria

Articles were selected based on the following inclusion criteria: (a) studies that involved any experimental research design, focusing on participants with LBP or related conditions, regardless of gender or age; (b) studies that investigated the effects of AH or AB techniques either as standalone interventions or in combination with other therapeutic approaches; (c) studies that compared the effectiveness of AH and/or AB techniques with any other intervention or with no intervention; (d) studies that reported outcomes relevant to trunk stability, muscle activation, pain reduction, functional performance, or rehabilitation outcomes; and (e) studies published in peer-reviewed journals and the English language. Studies were excluded if they lacked clear descriptions of the AH or AB interventions or were non-peer-reviewed sources such as conference abstracts, opinion papers, or editorials.

### 2.2. Information Sources and Search Strategy

The literature search was conducted in PubMed, Scopus, and PEDro, in July 2024. The search strategy employed combinations of terms related to LBP, AH, and AB techniques. For PubMed, we used the following string: *((“Abdominal Hollowing”[Title/Abstract] OR “Drawing-in Maneuver”[Title/Abstract] OR “Transversus Abdominis”[Title/Abstract] OR “Transversus Abdominis”[MeSH Terms] OR “Abdominal Bracing”[Title/Abstract] OR “Abdominal Contraction”[Title/Abstract] OR “Core Stabilization”[Title/Abstract] OR “Core Stability Training”[MeSH Terms]) AND (“Rehabilitation”[MeSH Terms] OR “Exercise Therapy”[MeSH Terms] OR “Physical Therapy Modalities”[MeSH Terms] OR “Exercise”[MeSH Terms] OR “Motor Control”[Title/Abstract])).* For Scopus, we restricted the string as follows: *(TITLE-ABS-KEY (“Abdominal Hollowing” OR “Drawing-in Maneuver” OR “Transversus Abdominis” OR “Abdominal Bracing” OR “Abdominal Contraction” OR “Core Stabilization” OR “Core Stability Training”) AND (TITLE-ABS-KEY (“Rehabilitation” OR “Exercise Therapy” OR “Physical Therapy Modalities” OR “Exercise” OR “Motor Control”).* Where search strings were not possible (i.e., PEDro database), selected individual keywords were used. Additionally, reference lists from relevant systematic reviews were manually searched to identify further studies. Finally, using all articles collected up to this point, a backward search (using reference lists) and forward search (using “cited by” function in Google Scholar) was performed. The records were managed using Mendeley (version 1.19.8) for duplicate removal, followed by an export to Microsoft Excel for screening and further analysis. The study search and selection process was conducted independently by the authors I.G. and M.O.Z., with verification by Z.K.

### 2.3. Data Extraction and Analysis

Data extraction was conducted independently by the authors I.G. and M.O.Z., with verification by Z.K. This approach process ensured that study selection was performed without bias and that only studies meeting the criteria were included in the review. Extracted data included (a) general study characteristics such as authors, publication year, sample size, and participant demographics; (b) details of the intervention including the type, frequency, and duration of AH and AB techniques; (c) characteristics of the control group, if applicable; and (d) outcome measures assessing trunk stability, muscle activation, pain, and functional performance pre- and post-intervention. Due to the significant heterogeneity in study populations, outcome measures, and intervention protocols across the included trials, a meta-analysis was not feasible. Thus, a narrative synthesis was employed to summarize the findings. Results were discussed among the authors to reach a consensus on subgroupings and interpretation of findings, ensuring comprehensive coverage of the research question. This approach allowed us to map the existing evidence on the use of AH and AB techniques in trunk stabilization and LBP rehabilitation, identifying gaps in the literature and areas requiring further research.

### 2.4. Study Quality Assessment

The quality of the included studies was assessed using the Physiotherapy Evidence Database (PEDro) Scale, which is a validated tool for evaluating the methodological rigor of clinical trials [22]. The PEDro Scale scores studies on 11 criteria, including random allocation, concealed allocation, baseline comparability, blinding (of subjects, therapists, and assessors), adequate follow-up, intention-to-treat analysis, between-group comparisons, and the reporting of point estimates and variability. The first criterion regarding eligibility criteria is not scored, resulting in a total score out of 10 for each study. Each study included in this review was independently assessed by two reviewers (I.G. and M.O.Z.), with discrepancies resolved through discussion or consultation with a third reviewer (Z.K.). The PEDro scores were then used to evaluate the overall methodological quality and risk of bias in the included studies. Studies scoring 6 or above were considered to have high methodological quality, while those scoring below 6 were categorized as having lower methodological quality. The assessment results were documented and are discussed in the context of interpreting the findings from the scoping review. A summary of the PEDro scores for each included study is provided in the Results section.

## 3. Results

### 3.1. Summary and General Study Characteristics

**Figure 1:**
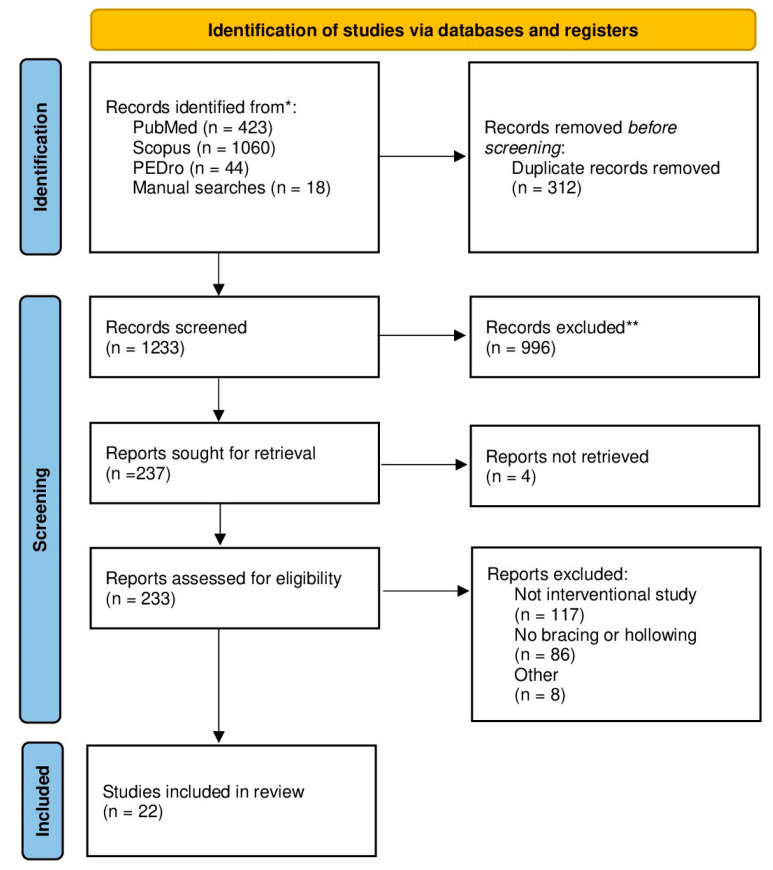
PRISMA flowchart of study search and selection. * reference listis of included papers were also screened; ** based on abstract and title screening.

In Table 1, data from a total of 22 Randomized Controlled Trials (RCTs) are presented. The flowchart of database search and study selection is shown in Figure 1. Of these, nine trials investigated both the AH and AB maneuvers [2,14,15,23,24,25,26,27,28]. Additionally, 11 RCTs focused solely on the AH maneuver [5,7,29,30,31,32,33,34,35,36,37] while 2 RCTs investigated only the AB maneuver [1,38].

Across all studies, the mean participant age was 37.7 ± 6.4 years. Specifically, 2 studies included exclusively female participants, 3 studies included exclusively male participants, and 17 studies included participants of both genders. In terms of inclusion criteria, 16 studies targeted individuals with acute, subacute, nonspecific, or chronic LBP. The remaining six studies focused on chronic stroke patients, healthy individuals, active individuals engaging in recreational sports regularly, or patients with chronic spinal cord injury.

The duration of interventions varied, with 8 weeks being the most common duration (n = 8), while 12 interventions were shorter (lasting between 20 days and 6 weeks), and 2 interventions were longer (ranging from 12 and 24 weeks). As for outcome measures, most authors assessed pain using VAS, NRS, or the McGill Pain Questionnaire. Additionally, disability was often measured using the ODI or RMDQ. Some studies also evaluated muscle thickness, functional tests, strength, balance, cross-sectional area, jump, vertical stiffness, pain, disability, muscle control, muscle activity, TMS (the excitability and organization of corticospinal inputs to TrA at the motor cortex), motor control, pulmonary function, activity limitation, or postural control. In the studies examined, various interventions were utilized across the experimental group or control group.

Interventions across the studies encompass a variety of approaches, including trunk stabilization exercises, lumbar stabilization exercises, core stability exercises, segmental stabilization, routine treatments, stabilization-enhanced general exercises, motor skill training, integrated training, motor control exercises, spinal stabilization rehabilitation programs, conventional lumbar dynamic strengthening exercises, specific exercises, manual treatments, and educational interventions. In comparing AB and AH interventions, it is noted that incorporating AH and AB maneuvers into trunk stabilization exercises, alongside conventional rehabilitation programs, led to significant enhancements in abdominal muscle thickness, balance, and gait measures [27]. Both AH and AB lumbar stabilization exercises demonstrated effectiveness in improving trunk strength and lower back impairment, particularly in individuals with insufficient trunk muscle power [15]. Additionally, AH core stability exercises showed significant improvements in muscle performance variables compared to AB exercises [23]. Moreover, segmental stabilization exercises effectively alleviated pain and disability, with notable enhancements in TrA activation [24]. The superiority of DMST over conventional methods was highlighted in reducing pain and improving functionality among individuals with NSLBP [2]. Stabilization-enhanced general exercises resulted in immediate greater improvement in self-reported impairment compared to general exercises alone. Specific exercises targeting local stabilizing muscles showed lower rates of LBP recurrences compared to medical management techniques over the long term [25]. For studies investigating AB and AH separately [35,37], improvements in spinal strength, function, and pain management were demonstrated for AB exercises, particularly emphasizing the effectiveness of isometric co-contractions and spinal stability exercises. AH exercises showed effectiveness in reducing disability, enhancing selective muscle contraction, improving respiratory function, and inducing neural reorganization in the motor cortex.

### 3.2. Study Quality Assessment

The assessment of study quality using the PEDro Scale revealed a range of methodological rigor across the included studies. Total PEDro scores varied from 3 to 7, indicating a spectrum from lower to higher quality. Notably, blinding of therapists and subjects was consistently underreported or absent, which may introduce bias. The details are presented in Table 2.

### 3.3. Studies Comparing AB and AH

In a study by Lee et al. [27], AH and BH were incorporated with trunk stabilization exercises, along with conventional rehabilitation programs, and led to statistically significant improvements in abdominal muscle thickness compared to controls. These interventions also led to notable enhancements in balance and gait measures post-intervention. Similarly, Kim et al. [15] explored the effectiveness of AH and AB combined with lumbar stabilization exercises in older women with NSLBP. Both groups demonstrated increased trunk strength and improved lower back impairment, particularly for individuals with insufficient trunk muscle power. Study Koh et al. [14] explored isolated AB and AH exercises targeting abdominal muscle activation, showcasing significant changes in muscle cross-sectional areas for both groups. Furthermore, isolated AH exercise in the study by Dupeyron et al. [23] exhibited statistically significant improvements in muscle performance variables when compared to the AB group, performing dynamic and static exercises such as side bridge, curl up, and pelvic bridging. Segmental stabilization exercises, focused on the LM an TrA muscles, and superficial strengthening exercises, focused on RA, OI, OE, and ES muscles, as examined in the study by França et al. [24], effectively alleviated pain and disability in NSLBP patients. Notably, segmental stabilization led to significant enhancements, including increased TrA activation. The studies by Kumar et al. [2] and Kumar et al. [26] underscored the superiority of DMST over conventional methods in reducing pain and improving functionality among NSLBP patients. In the DMST, AB and AH are integrated through progressive stages over a five-week period. Initially, patients are taught to perform AB (actively contracting the abdominals laterally) or AH (drawing the lower abdomen inward) while in a supine position. They then gradually progress to static stabilization under load, controlled movement, and finally, high-speed phasic exercises in various positions to optimize lumbar stabilization. In the study by Marshall et al. [28], the comparison between segmental and conventional exercises revealed that segmental exercises resulted in less impairment and decreased pain, although functional balance assessment scores did not statistically significantly differ between the groups. Lastly, the study by Koumantakis et al. [25] demonstrated statistically significant improvements in both groups following stabilization-enhanced general exercises, with immediate greater improvement in self-reported impairment observed in the general exercises group.

Overall, these studies underscore the effectiveness of incorporating AB and AH maneuvers into rehabilitation programs for improving trunk stability, reducing pain, and enhancing functional outcomes in individuals with various lower back conditions. Based on the analysis of various studies comparing AB and AH, there does not appear to be a clear superiority of one over the other. Studies such as Dupeyron et al. [23] demonstrate statistically significant improvements with isolated AH exercises compared to the AB group, especially in dynamic and static exercises like side bridge, curl up, and pelvic bridging. However, other studies like Koh et al. [14] have shown statistically significant changes in muscle cross-sectional areas for both AB and AH exercise groups. In summary, there is no definitive conclusion from existing research on which method is superior, thus suggesting the need for an individualized approach based on the needs and goals of each patient. However, Koh et al. [14] reported that AB, which can fully contract both deep and superficial muscles, is more effective for abdominal muscle activation than AH, which alone only contracts deep muscles; while Lee et al. [27] showed that AH was effective in improving TrA contraction and AB was effective in IO contraction in hemiplegic patients.

### 3.4. Studies Investigating AB

The study by Tayashiki et al. [38] demonstrated significant improvements in isometric trunk extension, hip extension, and maximum lifting power in the training group compared to the CG. Additionally, the training group exhibited increased rates of IAP rise during lifting tasks, maximal IAP during AB, and thickening of the OI muscle, whereas the CG did not show these improvements. For eight weeks, the training group trained three days a week for two seconds of maximal AB and two seconds of muscular relaxation (five sets of ten repetitions each day).

A study by Park et al. [1] consisted of two groups: the AB group, which incorporated AB into each session and applied it during spinal stability exercises, and the control group, which exclusively performed spinal stability exercises. Both groups showed increases in LLA over time, with no statistically significant difference between them. Both groups also experienced improvements in spine extensor strength, particularly at certain spinal flexion angles. However, the group undergoing AB exercises demonstrated greater improvements in pain and function compared to the CG. Additionally, spinal stabilization exercises led to changes in LLA among LBP patients.

In summary, both studies suggest that targeted exercises involving AB can lead to improvements in spinal strength, function, and pain management. Specifically, Tayashiki et al. [38] highlighted the effectiveness of isometric co-contractions in enhancing trunk and hip extension strength, while Park et al. [1] emphasized the benefits of spinal stability exercises, particularly AB, in improving pain and function outcomes in patients with LBP.

### 3.5. Studies Investigating AH

The study by Takasaki and Kawazoe [36] demonstrated that AH exercises with instantaneous feedback using ultrasonic imaging devices (Miruco) led to a statistically significantly lower ODI in the IG compared to the CG. This effect was observed over the course of one week of intervention followed by another week without intervention, therefore, we can assume that the effect is acute. However, the advantage of using the Miruco in enhancing isolated control of the TrA muscle during AH was limited. In the study by Lee et al. [29], AH with real-time ultrasound visual feedback resulted in a considerably larger ratio of RMS values of TrA-OI/OE muscles compared to conventional feedback methods. This indicated selective contraction of TrA-OI muscles versus the OE muscle. However, both groups showed no discernible difference in resting muscle thickness among TrA, internal OI, and OE muscles. In the study by Morales et al. [5], the authors found that proprioceptive Stabilizer™ training led to significant changes in abdominal wall muscle thickness, with increased thickness of TrA and decreased thickness of OI and OE muscles. This suggests potential benefits for individuals with lumbopelvic and LBP through proprioceptive stabilization training. In the study by Tsao et al. [37], motor skill training involving isolated voluntary contractions of the TrA induced neural reorganization in the motor cortex, which was particularly beneficial for individuals with recurrent pain. This suggests that motor training can reorganize neural networks in the motor cortex, potentially improving postural activation and functional outcomes. The study by Kim et al. [32] compared integrated training combining respiratory muscle training with AH maneuver exercises to respiratory muscle training alone and regular care. Integrated training resulted in significant improvements in FVC and FEV1, highlighting the synergistic effects of combining different therapeutic modalities. Akbari et al. [30] compared motor control exercises focusing on isometric low-load activation of local stabilizing muscles to general exercise activating paravertebral and abdominal muscles. Both groups showed statistically significant increases in muscle thickness and decreases in activity limitation, emphasizing the importance of targeted exercise interventions in improving functional outcomes. Both groups showed increases in muscle thickness and decreases in activity limitation over 8 weeks of training. Spine stabilization exercises in the study by Rhee et al. [35] led to statistically significantly reduced pain and impairment compared to medical management techniques. This was associated with significant differences in anterior/posterior sway, indicating improved postural control and reduced risk of injuries. Rasmussen-Barr et al. [34] evaluated a graded exercise intervention versus daily walks, showing long-term improvements in perceived disability, physical health, and self-assessment favoring the exercise group. This underscores the benefits of structured exercise programs in promoting long-term functional gains. Goldby et al. [31] compared a spinal stabilization rehabilitation program (including AH to selectively train the TrA muscle) to manual treatment and education. This highlights the effectiveness of targeted rehabilitation strategies in managing LBP. The study by Moon et al. [33] compared conventional lumbar dynamic strengthening exercises to lumbar stabilization exercises, with the latter showing statistically significantly greater improvements in lumbar extension strength and disability scores. This suggests the superiority of stabilization exercises in promoting functional recovery. Lastly, the study by Hides et al. [7] compared specific exercises targeting LM muscle in co-contraction with the TrA muscle to medical management techniques, showing lower rates of LBP recurrences in the exercise group over one to three years of follow-up. This underscores the importance of specific exercise interventions in preventing recurrent LBP.

In conclusion, findings from all these studies suggest that targeted exercises, including AH, can lead to improvements in spinal strength, function, and pain management. Various methods, such as feedback devices, proprioceptive training, and stabilization exercises, have been highlighted as effective approaches for addressing spinal issues. The importance of structured exercise programs and targeted rehabilitation approaches is underscored as crucial for long-term improvements in functional outcomes and quality of life in individuals with LBP.

## 4. Discussion

The aim of our study was to synthesize existing research to provide insights into the practical application of AH and AB techniques and their implications for clinical practice and rehabilitation strategies aimed at managing LBP. The analyzed research, including 22 randomized controlled trials, highlights the effectiveness of two different trunk stabilization techniques, AH and AB, in the treatment of LBP. The included studies suggest that these techniques are beneficial in improving muscle thickness, balance, gait, and other functional outcomes, but there is no clear superiority in efficacy between them.

### 4.1. Effects of the AH Manoeuvre

Evidence suggests that AH exercises, often supported by visual feedback mechanisms such as ultrasound, increase selective activation of these muscles, which is crucial for stabilizing the lower spine. Studies by Lee et al. [29] and Takasaki and Kawazoe [36] suggest that AH can lead to improvements in muscle performance, functional outcomes, and reductions in disability scores.

The selective engagement of TrA during AH, as highlighted by studies by Lee et al. [29] and Takasaki and Kawazoe [36], is helpful for stabilizing the lumbar spine without significantly increasing the intra-abdominal pressure, which can be particularly beneficial in the early stages of rehabilitation. The activation of TrA during AH has been linked to improved segmental control and reduced shear forces on the spinal segments, contributing to beneficial outcomes in pain reduction and functional mobility, likely due to the precision in muscular engagement and the subsequent decrease in mechanical stress on the lumbar spine [23].

Moreover, the neurological effects of these exercises add another layer to their benefits. For example, AH exercises have been associated with neural reorganization in the motor cortex, which can enhance postural control and potentially reduce the recurrence of pain by improving neural control over spinal stability, as suggested by Tsao et al. [37]. This indicates that the benefits of AH might not just be mechanical but also neuromuscular, enhancing the body’s internal coordination and control mechanisms.

### 4.2. Effects of the AB Manoeuvre

Research, such as studies by Koh et al. [14] and Tayashiki et al. [38], shows that AB can effectively increase trunk and hip strength, enhance muscle cross-sectional areas, and improve overall spinal stability. In particular, the study by Park et al. [1] underscores the benefit of incorporating AB into spinal stability exercises, which produced better outcomes in pain and function compared to control groups performing stability exercises alone.

### 4.3. Effects of AH, AB Maneuver, and/or Other Interventions

Several studies have demonstrated specific benefits associated with either AH or AB maneuvers when integrated or combined with other interventions. For example, Kumar et al. [2,26] found that dynamic motor stabilization training, which integrates both AH and AB, significantly reduced pain and improved functionality among individuals with non-specific LBP. Similarly, França et al. [24] highlighted that segmental stabilization exercises, which likely include elements of AH, effectively alleviated pain and disability, showing enhanced activation of TrA.

In studies including AH in the exercise program—by Morales et al. [5]; Lee et al. [29]; Akbari et al. [30]; Goldby et al. [31]; Kim et al. [32]; Moon et al. [33]; Rasmussen-Barr et al. [34]; Rhee et al. [35]; Tsao et al. [37]; and Hides et al. [39]—improvements were noted in muscle thickness, functional tests, and disability metrics, underscoring the utility of AH in different rehabilitation settings. These studies often utilized specific measurements like muscle thickness, pain scales, and functional disability assessments to gauge the effectiveness of interventions. In addition, one study, by Lee et al. [27], found that incorporating both AH and AB with trunk stabilization exercises led to significant improvements in abdominal muscle thickness and balance, highlighting the potential benefit of combining these techniques in rehabilitation programs. The evidence supports the integration of both AH and AB exercises into rehabilitation programs for LBP. These exercises not only improve core stability and muscle activation but also contribute significantly to pain management and functional recovery. This suggests that rehabilitation programs should be tailored to include a combination of these exercises, based on individual assessments and the specific needs of patients.

### 4.4. Comparison of AH and AB Maneuvers

When comparing AH and AB, no clear superiority of one technique over the other was evident across the studies. Both techniques have shown effectiveness in various domains such as muscle activation, pain reduction, and functional improvement.

The differences in muscle activation between AB and AH exercises and their implications in managing LBP are significant, driven largely by the biomechanical targets of each maneuver. The AB co-activation pattern provides a more generalized and robust enhancement of trunk stiffness, which is advantageous for overall spinal stability. This approach is especially beneficial in tasks requiring high levels of load transfer or spinal stability, as it prepares the body to handle substantial stresses, which could explain its effectiveness in improving trunk and hip strength [14,38].

The variability in findings across different studies can be attributed to several factors including the population studied, the specific protocol used, and the outcome measures prioritized. For instance, studies involving athletes or individuals with higher physical demands might find AB more beneficial due to its broad muscle recruitment patterns, whereas AH might be more suited for clinical populations or during the initial stages of rehabilitation. The context of the application, therefore, plays a crucial role in determining the effectiveness of each method, highlighting the need for a personalized approach in clinical practice based on individual assessments, the specific needs of patients, and the functional demands of their daily activities [2,26,27].

### 4.5. Summary and Clinical Applicability

In summary, both AB and AH maneuvers offer distinct advantages for managing LBP through different mechanisms of muscle activation and neuromuscular control. Understanding these underlying mechanisms helps in tailoring rehabilitation programs that are more effective and suited to the needs of individual patients, thereby optimizing outcomes in the treatment of lower back disorders. Our findings could guide work in the clinical setting to design more tailored therapeutic approaches for the treatment of patients with LBP. For example, selective muscle activation achieved using AH would be beneficial for patients in the early stages of rehabilitation or those with specific vulnerabilities, as it improves segmental control and reduces mechanical stress on the spine. In clinical rehabilitation programs, it would be reasonable to combine AH and AB maneuvers to increase trunk stability, reduce pain, and improve functional mobility and quality of life of patients.

### 4.6. Limitations

The studies present several shared limitations that should be considered when interpreting their results. Many of these studies, e.g., Morales et al. [5]; Kim et al. [15]; and Lee et al. [27], have noted methodological constraints such as small sample sizes, the absence of control groups, and a lack of long-term follow-up, which may limit the generalizability of their findings. Furthermore, the studies by França et al. [24] and Rhee et al. [35] did not incorporate intermediate or long-term evaluations or a comprehensive assessment of biopsychosocial factors, which could influence outcomes. The recruitment and participant selection also posed issues, for instance, Kim et al. [15] included only women, and Takasaki and Kawazoe [36] studied a cohort biased towards young Japanese university students, limiting wider applicability. Additionally, studies by Lee et al. [29] and Moon et al. [33] mentioned technological and measurement limitations such as the use of surface EMG instead of more accurate measures like fine-wire EMG or ultrasound, restricting precise muscle activity assessment. These common limitations highlight the need for future research with more robust designs, larger and more diverse populations, and more comprehensive measurement techniques to better understand the effectiveness and applicability of AH and AB exercises in various settings. The heterogeneity of the included studies, including variations in sample sizes, participant demographics (e.g., studies focusing solely on women or small, specific populations), and differences in the presence of control groups, limits the generalizability of the findings. Consequently, the conclusions of this review should be viewed as trends observed across diverse contexts rather than definitive statements applicable to all populations. This limitation underscores the need for more standardized research with larger, more diverse samples and consistent methodologies to strengthen the evidence base and provide clearer guidance for clinical practice. In addition, the heterogeneity of the included studies, particularly in terms of populations, outcomes, and intervention protocols, precluded a meta-analytic approach and limit the ability to generalize the findings quantitatively. Finally, we restricted the search to studies published in English, which could introduce language bias. However, we believe that the core body of literature on AH and AB is well-represented in English-language journals. Future studies might benefit from including studies in other languages to further reduce language bias.

### 4.7. Future Research

A critical evaluation of the findings reveals conflicting results across studies, particularly in the relative effectiveness of AH and AB for different populations and conditions. For instance, some studies indicate that AH leads to superior muscle activation in targeted deep muscles like the transversus abdominis, while others suggest that AB, through co-contraction of both deep and superficial muscles, provides better overall trunk stability. These inconsistencies may stem from variations in participant characteristics, such as differences in age, activity level, or the presence of specific conditions like chronic lower back pain, as well as disparities in the intervention protocols used, including the frequency and duration of the exercises.

Given these conflicting findings, future research should prioritize the following areas: (1) conducting head-to-head comparisons of AH and AB in more standardized settings, with consistent methodologies, to clarify their relative benefits across different populations; (2) investigating the long-term effects of both techniques, as most studies have focused on short-term outcomes; and (3) exploring how patient-specific factors, such as baseline muscle strength, motor control, and the severity of lower back pain, influence the effectiveness of each technique. Addressing these gaps will help to resolve current discrepancies and provide more reliable evidence to guide clinical decision-making.

## 5. Conclusions

In conclusion, our comprehensive review of 22 randomized controlled trials offers critical insights into the practical applications and efficacy of the AH and AB techniques. However, due to the significant heterogeneity in study populations, methodologies, and outcomes, the findings should be interpreted as indicative trends rather than definitive conclusions. Further research with standardized methodologies and larger, more diverse populations is needed to establish clearer evidence and provide stronger clinical recommendations for the use of AH and AB in rehabilitation settings.

## Figures and Tables

**Table 1:** Overview of the included studies.

Author (Year)	Participants			Interventions	Program Duration and Frequency	Outcome Measures	Results
	Sample Size	Age (Years) Mean ± SD	Other Characteristics				
Lee et al. (2020) [27]	n = 30 EG1: n = 10 EG2: n = 10 CG: n = 10	EG1: 66.89 ± 10.00 EG2: 69.57 ± 11.75 CG: 68.57 ± 9.54	Chronic stroke patients	**EG1: AH maneuver + conventional rehabilitation program** Trunk stabilization exercise with AH maneuver + conventional rehabilitation program (same as in CG). **EG2: AB maneuver + conventional rehabilitation program** Trunk stabilization exercise with AB maneuver + conventional rehabilitation program (same as in CG). **CG: Conventional rehabilitation program** Routine physical therapy and occupational therapy and usual care.	For trunk stability exercises: 20 min per session, 3 times per week, for 6 weeks	Abdominal muscle thickness; standing stability (FRT); dynamic balance (BBS); performance of gait (TUG); 10 MWT	Abdominal muscle thickness significantly changed in EG1 and EG2 compared to CG (*p* < 0.05). The values of the balance and gait measures, BBS, FRT, 10 MWT, and TUG, improved significantly (*p* < 0.05) after the intervention periods, although there were no significant differences between groups in the scores of the gait and balance scales.
Kim et al. (2018) [15]	n = 38 EG1: n = 17 EG2: n = 21	EG1: 70.6 ± 1.7 EG2: 66.8 ± 4.4	Older adult women with NSLBP	**EG1: AH lumbar stabilization exercise** Five lumbar stabilization exercises, including side plank exercise, bridge exercise, 4-kneeling exercise, prone plank exercise, and prone back extension exercise with AH maneuver. **EG2: AB lumbar stabilization exercise** Five lumbar stabilization exercises, including side plank exercise, bridge exercise, 4-kneeling exercise, prone plank exercise, and prone back extension exercise with AB maneuver.	1 h per session, 3 times per week, for 12 weeks	Trunk strength, low back disability (ODI), RMDQ, static balance (1-leg standing test)	According to this research, older adult women with NSLBP may benefit from HLSE and BLSE in community settings to increase their trunk strength and lower back impairment. In particular, older women with NSLBP who have insufficient trunk muscle power and a lower back impairment may benefit from HLSE and BLSE.
Koh et al. (2014) [14]	n = 30 EG1: n = 15 EG2: n = 15	EG1: 39.0 ± 5.4 EG2: 37.5 ± 3.4	Healthy, middle-aged women	**EG1: AB exercise** AB (1)-breathe in and out, AB (2)-supine position, plank exercise, side plank exercise. **EG2: AH exercise** Abdominal draw-in maneuvers in hook-lying position, standing position, sitting position, and in 4-point kneeling position.	1 h per session, 3 times per week, for 6 weeks	Cross-sectional area of TrA, OI, OE, and RA (CT)	Following the AB exercise, the left RA and both the OI and OA displayed statistically SSD (*p* < 0.05) in cross-sectional areas. Following the AH exercise, the left rectus abdominis and right transversus abdominis displayed SSD in cross-sectional areas (*p* < 0.05).
Dupeyron et al. (2013) [23]	n = 14 EG1: n = 7 EG2: n = 7	EG1: 19.1 ± 0.6 EG2: 18.1 ± 0.4	Male soccer players (national level) who had never experienced back pain or lower limb surgery	**EG1: AB core stability exercises** Curl up, side bridge, and pelvic bridging. **EG2: AH core stability exercises** Correct TrA activation, while lying in a supine position (15 series of 4 contractions, lasting 15 s), TrA strengthening with lower limb movements.	For AB: 1 min for 5 repetitions during 12 series, 2 times per week for 8 weeks. For AH: 8-week protocol divided into 2 sessions of 4 weeks	Contact time, flight time, jump height, leg stiffness (during hopping task-2.2 Hz)	While there was no change (*p* > 0.1) between the pre and post tests for the AB group, the abdominal strengthening therapy significantly improved all dependent variables (leg stiffness, *p* = 0.02; contact time, *p* = 0.02; flight duration, *p* = 0.02; jump height, *p* = 0.04) for the AH group. For every dependent variable, there was no change between the groups prior to and following training.
França et al. (2010) [24]	n = 30 EG1: n = 15 EG2: n = 15	EG1: 42.07 ± 8.15 EG2: 41.73 ± 6.42	Individuals with chronic LBP	**EG1: Segmental stabilization** AH maneuver for strengthening of the TrA and LM in 4-point kneeling, dorsal decubitus with knee flexed, ventral decubitus, and co-contraction of LM and TrA in upright position. **EG2: Superficial strengthening** AB maneuver for strengthening of the RA, ES, OI, and OE. For RA in dorsal decubitus with knee flexed and trunk flexion, in dorsal decubitus and knee semi-flexed. For ES in ventral decubitus with trunk extension and OI, EO, and RA in dorsal decubitus and with knee flexed.	30 min per session, 2 times per week, for 6 weeks	Functional disability (ODI), pain (VAS, McGill), TrA muscle activation capability	Both therapies were successful in reducing pain and enhancing disability as compared to baseline (*p* < 0.001). Comparing members of the segmental stabilization group to those in the ST group, they showed substantial increases in all variables (*p* < 0.001), including TrA activation.
Kumar et al. (2009) [2]	n = 30 EG1: n = 15 EG2: n = 15	EG1: 23.40 ± 3.27 EG2: 24.07 ± 2.89	Middle-aged male hockey players with subacute or chronic LBP	**EG1: Conventional treatment ** Ultrasound, short-wave diathermy, and lumbar strengthening exercises (spinal extensor exercise and trunk extensor exercise). **EG2: DMST** In DMST group, they used AB maneuver, because they used co-contraction of deep abdominal muscles such as TrA and LM. First week: facilitation and isolation of target muscles. Second week: trunk stabilization training under static conditions of increased load. Third week: development of trunk stabilization during the lumbar spine’s slow and controlled movement. Fourth and fifth weeks: stabilization of lumbar spine during skilled and high-speed movement.	40 min pr day, every day for 35 days	Pain, functional ability (walking, standing, climbing)	The outcomes demonstrated that while both therapies are useful in managing LBP, DMST proved to be more successful than traditional therapy. When compared to standard treatment, DMST produced greater improvements in walking, stand-ups, climbing, and pain. Walking, stand-ups, climbing, and discomfort all improved significantly (*p* < 0.01) over time (days) in DMST compared to conventional treatment.
Kumar et al. (2010) [26]	n = 141 EG1: n = 69 EG2: n = 72	EG1: 35.83 ± 0.66 EG2: 34.36 ± 0.72	Male and female patients with LBP	**EG1: Conventional treatment** Ultrasound, short-wave diathermy, and lumbar strengthening exercises (prone lying leg, chest elevation, and supine lying bridging). **EG2: DMST** In DMST group, they used AB maneuver because they used co-contraction of deep abdominal muscles such as TrA and LM. First week: facilitation and isolation of target muscles. Second week: trunk stabilization training under static conditions of increased load. Third week: development of trunk stabilization during the lumbar spine’s slow and controlled movement. Fourth and fifth weeks: stabilization of lumbar spine during skilled and high-speed movement.	40 min per day, every day for 20 days, with 180 day follow-up	Pain, functional ability (walking, ascending stairs, and stand-ups), physical strength (BPC, APC), and QOL	The study reports improvements in pain, BPC, APC, walking, stair climbing, and stand-ups for both genders in DMST compared to conventional. Furthermore, QOL improved more in DMST for female participants than in conventional group.
Marshall et al. (2013) [28]	n = 64 EG1: n = 32 EG2: n = 32	EG1: 36.2 ± 8.2 EG2: 36.2 ± 6.2	Patients with chronic nonspecific LBP	**EG1: SEG** Skilled abdominal contractions and postural training included AB and AH maneuvers. AH for isolation and during inner core exercises in side lying trunk exercise (mat-based), prone lying trunk exercise (mat and reformer training), hip-specific exercise (mat and reformer training, and upper and lower limb exercise (reformer based). They also used biofeedback pressure transducer under the lumbar spine. AB maneuver was used during neutral spine posture and full body exercise. **EG2: CEG** Warm-up and whole body stretching, specific cycle technique, seated hill type, flat road cycling, mixed resistance work, sprint focus and warm down with whole body stretching.	50–60 min per day, 3 times per week, for 8 weeks	Pain (VAS), disability (ODI), catastrophizing (PCS), FAB (FABQ)	At eight weeks, the SEG’s impairment was considerably less than the CEG’s (*p* = 0.018). Following training, pain decreased from baseline in both groups (*p* < 0.05), but it was less for the SEG (*p* < 0.05). FAB scores decreased in the CEG at six months and in the SEG at eight weeks. There were no differences in FAB scores across the groups.
Koumantakis et al. (2005) [25]	n = 55 EG1: n = 29 EG2: n = 26	EG1: 39.2 ± 11.4 EG2: 35.2 ± 9.7	Patients with recurrent, nonspecific LBP	**EG1: Stabilization-enhanced general exercise** AH was used in low-load activation of local stabilizing muscles (isometrically) and minimum loading positions (supine lying, 4-point kneeling, sitting, and standing). AB was used in whole-body exercises. **EG2: General exercise-only** Exercises activating the extensor (paraspinals) and flexor (abdominals) muscle groups.	45–60 min per day, 2 times per week, for 8 weeks	Pain (McGill), disability (RMDQ), cognitive status (Tampa scale of kinesiophobia, Pain locus control scale).	Both groups’ outcome measures showed improvement. Additionally, the general exercise-only group saw a greater improvement in self-reported impairment immediately following the intervention, but not at the 3-month follow-up. For all other outcomes, there were no significant differences seen between the two exercise programs.
Tayashiki et al. (2016) [38]	n = 20 EG: n = 11 CG: n = 9	EG: 23.5 ± 2.0 CG: 23.1 ± 1.9	Young, active men who regularly participate in recreational sports	**EG: Training group** AB was used In neutral lumbar spine sitting position (2 s maximal isometric co-contractions) and 2 s muscle relaxation with 2 min intervals between sets. Participants were asked to maximally contract abdominal muscles without changing upper body position and without hollowing the lower abdomen. **CG: Measurements** Maximal voluntary isometric strength during trunk flexion and extension, hip extension, and knee extension, maximal lifting power from sitting position, and the thicknesses of abdominal muscles.	For EG: about 12 min, 3 days per week, for 8 weeks.	Strength, muscle thickness	While CG did not exhibit any improvements in strength and power measures, training group demonstrated substantial gains in isometric trunk extension, hip extension, and maximum lifting power following the intervention. In addition, training group significantly increased the rate of IAP rise during lifting tasks, maximal IAP during abdominal bracing, and the thickness of the oblique internal muscle without affecting CG.
Park et al. (2023) [1]	n = 67 EG: n = 33 CG: n = 34	EG: 44.8 ± 10.8 CG: 43.0 ± 10.6	Patients with nonspecific chronic LBP	**EG: AB spinal stability exercises** Preparation phase (patient in supine position with knees bent and towel underneath the participant’s torso), relaxation phase (the participant pushes therapist’s hand on his abdomen while keeping lower back free from the floor and contraction phase (participant maintains abdominal pressure, while therapist pulls the towel, to prevent the towel from coming out). **CG: Spinal stability exercises** Early phase (0–8 weeks) on stable ground, intermediate phase (8–16 weeks), and late phase (16–24 weeks) on unstable ground using gym ball or balance pad.	50 min per day, 2 times per week, for 24 weeks.	Pain (VAS), disability (ODI).	Over time, both groups’ LLA increased, but there was no discernible difference in LLA between them. Both groups’ spine extensor strength increased over time, and at 60° and 72° spinal flexion angles, an interaction effect was seen. Over time, both groups experienced improvements in pain and function; however, the ABBG group experienced a greater improvement than the control group. Spinal stabilization exercises altered the LLA in CLBP patients.
Takasaki and Kawazoe (2021) [36]	n = 60 EG1: n = 20 EG2: n = 20 CG: n = 20	EG1: 21.0 ± 3.7 EG2: 19.1 ± 1.2 CG: 20.3 ± 4.9	Patients with LBP	EG1: AH with miruco Workout of the AH with instantaneous feedback through the use of the ultrasonic imaging device, the Miruco. EG2: AH with self-palpation At-home exercises for AH with self-palpation of the TrA and OI, OE muscles. CG: Wait and see	For EG1 and EG2: 2 courses, 3 sets, and 10 repetitions of the AH per day for 1 week	Muscle thickness, disability (ODI).	Consequently, for every follow-up period, there was not a statistically significant interaction effect (*p* > 0.05) in the changes in the primary outcome measures from baseline. Following the intervention, the abdominal H with Miruco group’s ODI was statistically lower (*p* = 0.036) than that of the control group. The results show that using the Miruco in abdominal H home exercise to enhance isolated control of the TrA muscle during AH has a limited advantage.
Lee et al. (2018) [29]	n = 20 EG1: n = 10 EG2: n = 10	EG1+ EG2: 29.00 ± 3.00	Healthy adults without LBP	**EG1: AH using conventional feedback** Lying in supine position, subjects placed fingers 2 cm medial and caudal to the SIAS and felt contractions. Physiatrist confirmed if the subject had positioned their fingertips correctly. **EG2: AH visual feedback supplemented by real-time ultrasound imaging** The individual was instructed to cough prior to the AH in order to demonstrate movement of their abdominal muscles on the monitor. After that, the participant did the AH with real-time visual input while staring at the display. The movements included lateral movement and thickness of the TrA, thickening of the OI, and avoiding contraction of the OE muscle.	20 min per day, 3 times per week, for 2 weeks	Muscle thickness (TrA, OI, OE).	In both groups, there was no discernible difference in the resting muscle thickness of TrA, OI, and OE. Nonetheless, EG2 exhibited a considerably larger ratio of RMS values of TrA-OI/OE muscles compared to group EG1. This ratio indicates the selective contraction of TrA-OI muscles versus OE muscle.
Morales et al. (2018) [5]	n = 41	EG: 31.9 ± 4.5	Healthy adults	At rest and during the AH, the measurements were taken. Using a circular pressure marker as a visual stimulus, the patients held 40 mmHg for 10 s while wearing the StabilizerTM in the lower back.	/	Muscle thickness (TrA, OI, OE, RA).	The thickness decreased for the OE and OI and the thickness rise of TrA were statistically significant changes (*p* < 0.05) in the abdominal wall muscles measured by ultrasound. In healthy volunteers, proprioceptive StabilizerTM training resulted in an increase in muscle thickness in the TrA muscle and a decrease in muscle thickness in the OE and OI muscles. These results imply that those with lumbopelvic and LBP may benefit from proprioceptive stabilization training.
Tsao et al. (2010) [37]	n = 20 EG: n = 10 CG: n = 10	EG: 24 ± 8 CG: 23 ± 3	Patients with recurrent LBP	**EG: Motor skill training** AH maneuver for isolated voluntary contractions of TrA to improve motor control and spinal stability. **CG: Self-paced walking exercise** Activation of TrA along with activation of other limb and trunk muscles.	For EG: 2 weeks For CG: 10 min twice per day, for 2 weeks	Muscle activity (TrA), TMS, motor control.	Anterior and medial shifts towards the motor cortex representation of TrA seen in healthy subjects were generated by motor skill training. This change was linked to early TrA postural activation. Following an inexperienced walking exercise, no changes were seen. This is the first evidence that individuals with recurrent pain can have their neural networks in the motor cortex reorganized in the opposite direction by motor training.
Kim et al. (2017) [32]	n = 37 EG1: n = 13 EG2: n = 13 CG: n = 12	EG1: 39.98 ± 11.47 EG2: 41.51 ± 10.04 CG: 40.12 ± 8.73	Patients with chronic spinal cord injury	**EG1: Integrated training ** Respiratory muscle training + AH maneuver exercise. **EG2: Respiratory muscle training** Respiratory muscle training. **CG: Regular care or alternate, routine physical therapy** Alternative and routine physical therapy and usual care.	Over 8 week period	Pulmonary function (FVC, FEV).	The FE1 and FVC pre- and post-test values differed significantly across the groups. Following the test, there were notable variations between the FVC and FEV1 values in the ITG, RMTG, and CG. Additionally, compared to the RMTG, the change ratio values of the FVC and FEV1 in the ITG increased by an average of 9.75% and 7.91%, respectively, after the 8-week intervention.
Akbari et al. (2008) [30]	n = 49 EG1: n = 25 EG2: n = 24	EG1: 39.6 ± 3.5 EG2: 40 ± 3.6	Patients with chronic LBP	**EG1: Motor control exercise** Activation of the TrA with AH maneuver. Isometric low-load activation of the local stabilizing muscles was applied in minimally loading poses (4-point kneeling, supine reclining, sitting, and standing). **EG2: General exercise** Activating paravertebral and abdominal muscles.	30 min per session (16-session exercise program), 2 times per week, for 8 weeks	Muscle thickness (TrA, LM), pain (VAS), activity limitation (Back Performance Scale)	The mean TA and LM thickness increased in the motor CG and the general exercise group (*p* < 0.0001). The mean activity limitation decreased in the motor CG and the general exercise group (*p* < 0.0001). After treatment, there was no SD between the two groups, with the exception of pain (*p* > 0.05).
Rhee et al. (2012) [35]	n = 42 EG: n = 21 CG: n = 21	EG: 53.09 ± 9.04 CG: 50.90 ± 5.24	Patients with LBP	EG: Spine stabilization exercises AH maneuver for co-contraction with the TrA muscle, activation, and training the isometric holding function of the spinal muscle at the affected vertebral segment. CG: Medical management techniques Bed rest, prescription medications, absence from work, and resuming normal activity.	5 times per week, for 4 weeks	Pain (VAS), disability (ODI), balance measurements	After both groups received treatment, there was a considerable reduction in the reported levels of pain and impairment. There was a significant difference between group and measurement duration during A/P sway (*p* = 0.04), even though the M/L sway was not statistically different in either group (*p* = 0.86). When compared to the control group, the SSE group’s A/P displacement dropped considerably. The SSE intervention can be connected to the decreased A/P displacement because it limits the rate at which an individual responds to outside disturbances, hence preventing more injuries.
Rasmussen-Barr et al. (2009) [34]	n = 71 EG1: n = 36 EG2: n = 35	EG1: 37 ± 10 EG2: 40 ± 12	Patients with nonspecific, recurrent LBP	**EG1: Graded exercise intervention** AH maneuver was used for specific exercises with instruction to draw in the anterolateral abdominal wall (TrA isolated from other muscles). **EG2: Daily walks** Walk at the fastest pace + home exercises.	For EG1: 1 h per session, for 8 weeks, with 6, 12 and 36 months follow-up For EG2: 30 min walk every day, for 8 weeks	Pain (VAS), disability (ODI)	At the 12-month follow-up, 83% of the participants gave data, and at the 36-month follow-up, 79% did so. A 12-month study comparing the two groups revealed a decline in perceived disability favoring the exercise group; however, no similar effect was seen for pain until right after the intervention. Long-term improvements were also seen in the exercise group’s assessments of physical health and self-efficacy beliefs; however, fear-avoidance beliefs did not change.
Goldby et al. (2006) [31]	n = 213 EG1: n = 84 EG2: n = 89 CG: n = 40	EG1: 43.4 ± 10.7 EG2: 41 ± 11.7 CG: 41.45 ± 13	Patients with chronic LBP	**EG1: Spinal stabilization rehabilitation program** AH maneuver was used in functionally progressive workout program emphasising the global muscle substitution mechanisms while selectively retraining the TrA, LM, pelvic floor, and diaphragm muscles. **EG2: Manual treatment** Exercise or manual procedure within the remit of musculoskeletal physiotherapy. **CG: Education** Back in Action” booklet.	For EG1: 10, 1 h classes For EG2: maximum of 10 interventions	Pain (NRS), disability (ODI), handicap, QOL	The findings showed statistically significant improvements in favor of the spinal stabilization group in terms of medication, disability, and pain at the six-month and one-year stages.
Moon et al. (2013) [33]	n = 21 EG1: n = 10 EG2: n = 11	EG1: 28.6 ± 4.9 EG2: 28.4 ± 5.0	Patients with chronic LBP	The particular guidelines that guided all of the exercises were to breathe in and out and to slowly and softly draw in your lower abdomen below your umbilicus without moving your upper stomach, back, or pelvis. This is known as AH maneuver. **EG1: Conventional lumbar dynamic strengthening exercise** The flexor (rectus abdominis) and extensor (erector spinae) muscle groups were engaged by 14 exercises. **EG2: Lumbar stabilization exercise** There were 16 exercises designed to strengthen the TrA, LM, and OI.	1 h per session, 2 times per week, for 8 weeks	Muscle strength (lumbar extensor), pain (VAS), disability (ODQ)	After eight weeks, there was a significant improvement in both groups’ lumbar extension strength at all angles as compared to the baseline. At 0° and 12° of lumbar flexion, the lumbar stabilization exercise group showed noticeably bigger improvements. After therapy, the VAS dramatically fell, although there was no significant difference in the changes between the groups. Only in the group that performed stabilization exercises did ODQ scores considerably improve.
Hides et al. (2001) [39]	n = 39 EG: n = 20 CG: n = 19	EG: 31 ± 7 CG: 31 ± 8	Patients with first episode of LBP	**EG: Specific exercise** AH was used in training and activating the LM muscle’s isometric holding function at the affected vertebral segment (in conjunction with the TrA). Real-time ultrasound imaging revealed the multifidus contraction. **CG: Medical management techniques** Recommendations for bed rest, time off from work, medication prescriptions, and encouragement to return to regular activities.	2 times per week, for 4 weeks	Pain (McGill, VAS), disability (RMDQ)	According to questionnaire responses, patients in the targeted exercise group had a lower rate of LBP recurrences than patients in the CG. Recurrence in the specific exercise group was 30% and in the control group was 84% a year following treatment (*p* < 0.001). Specific exercise group recurrence was 35% and control group recurrence was 75% two to three years after therapy (*p* < 0.01).

n: numerus; EG: experimental group; CG: control group; AH: abdominal hollowing; AB: abdominal bracing; FRT: the Functional Reach Test; BBS: the Berg Balance Scale; TUG: the Timed Up and Go test; 10MWT: 10 m Walk test; NSLBP: nonspecific lower back pain; ODI: Oswerstry Disability Index; RMDQ: Roland–Morris Disability Questionnaire; TrA: transversus abdominis; LM: lumbar multifidus; RA: rectus abdominis; OE: abdominus obliquus externus; OI: rectus obliquus internus; ES: erector spinae; VAS: Visual Analog Scale; SIAS: spina iliaca anterior superior; DMST: Dynamic Muscular Stabilization Technique; QOL: quality of life; CEG: stationary cycling group; SEG: specialist trunk exercise group; FAB: fear-avoidance beliefs; PCS: Pain Catastrophizing Scale; FABQ: Fear-Avoidance Beliefs Questionnaire; IAP: intra-abdominal pressure; LLA: lumbar lordosis angle; RMS: root mean square; SCI: spinal cord injury; RMT: respiratory muscle training; FEV1: forced expiratory volume in 1 s; FVC: forced vital capacity; A/P: anterior–posterior; M/L: medio-lateral; ODQ: Ostry Low Back Pain Disability Questionnaire; SSE: spinal stabilization exercise; TMS: transcranial magnetic stimulation.

**Table 2:** Assessment of study quality with PEDro Scale.

	Total	Eligibility Criteria	Random Allocation	Concealed Allocation	Baseline Comparability	Blind Subjects	Blind Therapists	Blind Assessors	Adequate Follow-Up	Intention-to-Treat Analysis	Between-Group Comparisons	Point Estimates and Variability
Lee et al. [27]	6	1	1	0	1	0	0	1	1	0	1	1
Kim et al. [15]	4	1	0	0	1	0	0	0	1	0	1	1
Koh et al. [14]	3	1	0	0	1	0	0	0	0	0	1	1
Dupeyron et al. [23]	7	1	1	0	1	0	0	0	1	1	1	1
França et al. [16]	7	1	1	1	1	0	0	1	1	0	1	1
Kumar et al. [26]	4	1	1	0	0	0	0	0	1	0	1	1
Kumar et al. [2]	5	0	1	1	0	1	0	0	0	0	1	1
Marshall et al. [28]	7	1	1	1	1	0	0	0	1	1	1	1
Koumantakis et al. [25]	7	1	1	1	1	0	0	1	0	1	1	1
Tayashiki et al. [38]	5	1	1	0	1	0	0	0	1	0	1	1
Park et al. [1]	7	1	1	1	1	0	0	1	1	0	1	1
Takasaki and Kawazoe [36]	7	1	1	1	1	0	0	1	1	0	1	1
Lee et al. [29]	7	1	1	0	1	0	0	0	1	1	1	1
Morales et al. [5]	3	1	0	0	0	0	0	0	1	0	1	1
Tsao et al. [37]	7	0	1	1	1	0	0	1	1	0	1	1
Kim et al. [32]	6	1	1	0	1	0	0	1	1	0	1	1
Akbari et al. [30]	5	1	1	0	1	0	0	1	0	0	1	1
Rhee et al. [35]	5	1	1	1	1	0	0	0	0	0	1	1
Rasmussen-Barr et al. [34]	7	1	1	1	1	0	0	0	1	1	1	1

## Data Availability

No new data were generated for this paper (review).

## References

[1] Park H.S., Park S.W., Oh J.-K. (2023). Effect of Adding Abdominal Bracing to Spinal Stabilization Exercise on Lumbar Lordosis Angle, Extensor Strength, Pain, and Function in Patients with Non-Specific Chronic Low Back Pain: A Prospective Randomized Pilot Study. Medicine.

[2] Kumar S., Sharma V.P., Negi M.P.S. (2009). Efficacy of Dynamic Muscular Stabilization Techniques (DMST) over Conventional Techniques in Rehabilitation of Chronic Low Back Pain. J. Strength Cond. Res..

[3] Wu A., March L., Zheng X., Huang J., Wang X., Zhao J., Blyth F.M., Smith E., Buchbinder R., Hoy D. (2020). Global Low Back Pain Prevalence and Years Lived with Disability from 1990 to 2017: Estimates from the Global Burden of Disease Study 2017. Ann. Transl. Med..

[4] Studnicka K., Ampat G. (2024). Lumbar Stabilization. StatPearls.

[5] Morales C.R., Sanz D.R., Reguera M.d.l.C., Martínez S.F., González P.T., Pascual B.M. (2018). Proprioceptive Stabilizer^TM^ Training of the Abdominal Wall Muscles in Healthy Subjects: A Quasi-Experimental Study. Rev. Assoc. Medica Bras..

[6] Whittaker J.L., Warner M.B., Stokes M. (2013). Comparison of the Sonographic Features of the Abdominal Wall Muscles and Connective Tissues in Individuals with and without Lumbopelvic Pain. J. Orthop. Sports Phys. Ther..

[7] Hides J.A., Jull G.A., Richardson C.A. (2001). Long-Term Effects of Specific Stabilizing Exercises for First-Episode Low Back Pain. Spine.

[8] Barr K.P., Griggs M., Cadby T. (2005). Lumbar Stabilization: Core Concepts and Current Literature, Part 1. Am. J. Phys. Med. Rehabil..

[9] Barr K.P., Griggs M., Cadby T. (2007). Lumbar Stabilization: A Review of Core Concepts and Current Literature, Part 2. Am. J. Phys. Med. Rehabil..

[10] Smeets R.J.E.M., Vlaeyen J.W.S., Hidding A., Kester A.D.M., van der Heijden G.J.M.G., Knottnerus J.A. (2008). Chronic Low Back Pain: Physical Training, Graded Activity with Problem Solving Training, or Both? The One-Year Post-Treatment Results of a Randomized Controlled Trial. Pain.

[11] Hodges P.W., Richardson C.A. (1996). Inefficient Muscular Stabilization of the Lumbar Spine Associated with Low Back Pain. A Motor Control Evaluation of Transversus Abdominis. Spine.

[12] Hides J.A., Richardson C.A., Jull G.A. (1996). Multifidus Muscle Recovery Is Not Automatic after Resolution of Acute, First-Episode Low Back Pain. Spine.

[13] McGill S. (2015). Low Back Disorders: Evidence-Based Prevention and Rehabilitation.

[14] Koh H.-W., Cho S.-H., Kim C.-Y. (2014). Comparison of the Effects of Hollowing and Bracing Exercises on Cross-Sectional Areas of Abdominal Muscles in Middle-Aged Women. J. Phys. Ther. Sci..

[15] Kim M., Kim M., Oh S., Yoon B. (2018). The Effectiveness of Hollowing and Bracing Strategies with Lumbar Stabilization Exercise in Older Adult Women with Nonspecific Low Back Pain: A Quasi-Experimental Study on a Community-Based Rehabilitation. J. Manip. Physiol. Ther..

[16] França F.R., Burke T.N., Caffaro R.R., Ramos L.A., Marques A.P. (2012). Effects of Muscular Stretching and Segmental Stabilization on Functional Disability and Pain in Patients with Chronic Low Back Pain: A Randomized, Controlled Trial. J. Manip. Physiol. Ther..

[17] Maeo S., Takahashi T., Takai Y., Kanehisa H. (2013). Trunk Muscle Activities during Abdominal Bracing: Comparison among Muscles and Exercises. J. Sports Sci. Med..

[18] Aleksiev A.R. (2014). Ten-Year Follow-up of Strengthening versus Flexibility Exercises with or without Abdominal Bracing in Recurrent Low Back Pain. Spine.

[19] Kisner C., Colby L.A., Borstad J. (2017). Therapeutic Exercise: Foundations and Techniques.

[20] Himes M.E., Selkow N.M., Gore M.A., Hart J.M., Saliba S.A. (2012). Transversus Abdominis Activation during a Side-Bridge Exercise Progression Is Similar in People with Recurrent Low Back Pain and Healthy Controls. J. Strength Cond. Res..

[21] Tricco A.C., Lillie E., Zarin W., O’Brien K.K., Colquhoun H., Levac D., Moher D., Peters M.D.J., Horsley T., Weeks L. (2018). PRISMA Extension for Scoping Reviews (PRISMA-ScR): Checklist and Explanation. Ann. Intern. Med..

[22] Moseley A.M., Szikszay T., Lin C.-W.C., Mathieson S., Elkins M., Herbert R.D., Maher C., Sherrington C. (2015). A Systematic Review of the Measurement Properties and Usage of the Physiotherapy Evidence Database (PEDRO) Scale. Physiotherapy.

[23] Dupeyron A., Hertzog M., Micallef J.-P., Perrey S. (2013). Does an Abdominal Strengthening Program Influence Leg Stiffness during Hopping Tasks?. J. Strength Cond. Res..

[24] França F.R., Burke T.N., Hanada E.S., Marques A.P. (2010). Segmental Stabilization and Muscular Strengthening in Chronic Low Back Pain: A Comparative Study. Clin. Sao Paulo Braz..

[25] Koumantakis G.A., Watson P.J., Oldham J.A. (2005). Trunk Muscle Stabilization Training plus General Exercise versus General Exercise Only: Randomized Controlled Trial of Patients with Recurrent Low Back Pain. Phys. Ther..

[26] Kumar S., Sharma V.P., Shukla R., Dev R. (2010). Comparative Efficacy of Two Multimodal Treatments on Male and Female Sub-Groups with Low Back Pain (Part II). J. Back Musculoskelet. Rehabil..

[27] Lee J., Jeon J., Lee D., Hong J., Yu J., Kim J. (2020). Effect of Trunk Stabilization Exercise on Abdominal Muscle Thickness, Balance and Gait Abilities of Patients with Hemiplegic Stroke: A Randomized Controlled Trial. NeuroRehabilitation.

[28] Marshall P.W.M., Kennedy S., Brooks C., Lonsdale C. (2013). Pilates Exercise or Stationary Cycling for Chronic Nonspecific Low Back Pain: Does It Matter? A Randomized Controlled Trial with 6-Month Follow-Up. Spine.

[29] Lee D.H., Hong S.K., Lee Y.-S., Kim C.-H., Hwang J.M., Lee Z., Kim J.M., Park D. (2018). Is Abdominal Hollowing Exercise Using Real-Time Ultrasound Imaging Feedback Helpful for Selective Strengthening of the Transversus Abdominis Muscle?: A Prospective, Randomized, Parallel-Group, Comparative Study. Medicine.

[30] Akbari A., Khorashadizadeh S., Abdi G. (2008). The Effect of Motor Control Exercise versus General Exercise on Lumbar Local Stabilizing Muscles Thickness: Randomized Controlled Trial of Patients with Chronic Low Back Pain. J. Back Musculoskelet. Rehabil..

[31] Goldby L.J., Moore A.P., Doust J., Trew M.E. (2006). A Randomized Controlled Trial Investigating the Efficiency of Musculoskeletal Physiotherapy on Chronic Low Back Disorder. Spine.

[32] Kim C.-Y., Lee J.-S., Kim H.-D., Lee D.-J. (2017). Short-Term Effects of Respiratory Muscle Training Combined with the Abdominal Drawing-in Maneuver on the Decreased Pulmonary Function of Individuals with Chronic Spinal Cord Injury: A Pilot Randomized Controlled Trial. J. Spinal Cord Med..

[33] Moon H.J., Choi K.H., Kim D.H., Kim H.J., Cho Y.K., Lee K.H., Kim J.H., Choi Y.J. (2013). Effect of Lumbar Stabilization and Dynamic Lumbar Strengthening Exercises in Patients With Chronic Low Back Pain. Ann. Rehabil. Med..

[34] Rasmussen-Barr E., Ang B., Arvidsson I., Nilsson-Wikmar L. (2009). Graded Exercise for Recurrent Low-Back Pain: A Randomized, Controlled Trial with 6-, 12-, and 36-Month Follow-Ups. Spine.

[35] Rhee H.S., Kim Y.H., Sung P.S. (2012). A Randomized Controlled Trial to Determine the Effect of Spinal Stabilization Exercise Intervention Based on Pain Level and Standing Balance Differences in Patients with Low Back Pain. Med. Sci. Monit. Int. Med. J. Exp. Clin. Res..

[36] Takasaki H., Kawazoe S. (2021). Investigation on the Effectiveness of Abdominal Hollowing Home-Exercises Using a Portable Ultrasound: Randomized Controlled Trial. J. Electromyogr. Kinesiol. Off. J. Int. Soc. Electrophysiol. Kinesiol..

[37] Tsao H., Galea M.P., Hodges P.W. (2010). Driving Plasticity in the Motor Cortex in Recurrent Low Back Pain. Eur. J. Pain.

[38] Tayashiki K., Maeo S., Usui S., Miyamoto N., Kanehisa H. (2016). Effect of Abdominal Bracing Training on Strength and Power of Trunk and Lower Limb Muscles. Eur. J. Appl. Physiol..

[39] Hides J., Wilson S., Stanton W., McMahon S., Keto H., McMahon K., Bryant M., Richardson C. (2006). An MRI Investigation into the Function of the Transversus Abdominis Muscle during “Drawing-in” of the Abdominal Wall. Spine.

